# Evolution of a sexually dimorphic trait in a broadly distributed topminnow (*Fundulus olivaceus*)

**DOI:** 10.1002/ece3.242

**Published:** 2012-07

**Authors:** Jacob F Schaefer, David D Duvernell, Brian R Kreiser, Charles Champagne, Scott R Clark, Melissa Gutierrez, Laura K Stewart, Chazz Coleman

**Affiliations:** 1 Department of Biological Sciences, The University of Southern MississippiHattiesburg, Mississippi, 39406; 2 Department of Biological Sciences, Southern Illinois University EdwardsvilleEdwardsville, Illinois, 62026

**Keywords:** Geographic variation, sexual dimorphism, sexual selection, natural selection

## Abstract

Understanding the interaction between sexual and natural selection within variable environments is crucial to our understanding of evolutionary processes. The handicap principle predicts females will prefer males with exaggerated traits provided those traits are indicators of male quality to ensure direct or indirect female benefits. Spatial variability in ecological factors is expected to alter the balance between sexual and natural selection that defines the evolution of such traits. Male and female blackspotted topminnows (Fundulidae: *Fundulus olivaceus*) display prominent black dorsolateral spots that are variable in number across its broad range. We investigated variability in spot phenotypes at 117 sites across 13 river systems and asked if the trait was sexually dimorphic and positively correlated with measures of fitness (condition and gonadosomatic index [GSI]). Laboratory and mesocosm experiments assessed female mate choice and predation pressure on spot phenotypes. Environmental and community data collected at sampling locations were used to assess predictive models of spot density at the individual, site, and river system level. Greater number of spots was positively correlated with measures of fitness in males. Males with more spots were preferred by females and suffered greater mortality due to predation. Water clarity (turbidity) was the best predictor of spot density on the drainage scale, indicating that sexual and natural selection for the trait may be mediated by local light environments.

## Introduction

Sexual selection is known to be a potent evolutionary force. Understanding how various sexual selection mechanisms work within the context of variable environments is crucial to our understanding of evolutionary processes (Candolin and Heuschele 2008; Walsh and Reznick 2009; Ingleby et al. 2010). The handicap principle predicts that females will prefer males with exaggerated traits that are indicators of genetic quality (Iwasa et al. 1991; Iwasa and Pomiankowski 1999; Cotton et al. 2009). Male-exaggerated traits must be indicators of increased heritable fitness if females are to benefit indirectly through increased offspring fitness. The cost of exaggerated male traits may be extensive with natural selection mediating the trade-off between male fitness and expression of exaggerated traits (Rosenthal et al. 2001). Spatial and temporal environmental variability can alter this balance between sexual and natural selection, preventing populations from becoming fixed by persistent directional selection (Möller and Alatalo 1999; Sharma et al. 2010; Vergara et al. 2011). Local environmental conditions can dramatically affect the efficacy of sexual signals (Fuller and Travis 2004; Millar et al. 2006; Vergara et al. 2011) and survival rates of males expressing exaggerated traits (Houde and Endler 1990; Millar et al. 2006; Gordon et al. 2011). Empirical data on broadly distributed sexually dimorphic species found in a range of ecological conditions is needed to better understand the dynamics of natural and sexual selection within a meaningful ecological context.

Freshwater fish have served as model study systems for a number of fundamental questions in evolutionary ecology. A major advantage of these systems is that streams feature linear and predictable gradients in ecologically important biotic and abiotic factors (Vannote et al. 1980). In many cases, natural barriers within or among river systems form replicate evolutionary units. In Trinidad killifish (*Rivulus hartii*), predation pressure and resource availability correlated with stream gradients shape the evolution of a number of life-history traits (Fraser et al. 1995; Reznick et al. 2001; Walsh and Reznick 2009, 2010). Predation pressure and flow regimes influence the evolution of body shape and a number of correlates of performance in *Gambusia affinis* (Langerhans et al. 2004; Langerhans 2008; Langerhans and Reznick 2010). Studies of sexual and natural selection within an ecological context have shown female choice, light environments, and predation pressure to interact in driving the evolution of male phenotypes (Houde and Endler 1990; Endler 1993; Millar et al. 2006). Millar et al. (2006) linked the relative number of different color spots to water clarity and the physiological constraints of predator vision systems in contrasting environments. While these and other well-studied systems serve as foundations to our understanding of the evolutionary process, they are often limited in scope. Predation regimes are typically a dichotomous presence or absence and the distribution of the species studied are either limited or only studied in detail in a limited area (Krebs and Bell 2011). We combined laboratory experiments of mate choice and predation pressure with fieldwork conducted along stream gradients across 13 drainages to address questions regarding observed variability in the expression of a sexually dimorphic trait.

The genus *Fundulus* contains 38 extant species that inhabit a variety of habitats in North and Central America (Fuller et al. 2007; Whitehead 2010). The majority of species in the group are sexually dimorphic with individual species expressing a variety of sexually dimorphic traits such as vertical bars, lateral stripes, color patterns on the body or fins, and various color spotting patterns. Within the group, genetic and plastic (linked with water clarity and transmission of ultraviolet [UV]) effects influence vision systems and color phenotypes of *Lucania goodei* (Fuller and Travis 2004; Fuller et al. 2005). The group selected for this study is the widely distributed *Fundulus notatus* species complex, which occurs throughout most of the central United States. The *F. notatus* species complex is comprised of the Blackstripe Topminnow (*F. notatus*), the Blackspotted Topminnow (*F. olivaceus*), and the Broadstripe Topminnow (*F. euryzonus*) that all share similar niches (Thomerson and Woolridge 1970; Blanchard 1996) and hybridize in contact zones found throughout their ranges (Thomerson 1967; Setzer 1970; Duvernell et al. 2007; Schaefer et al. 2011). Throughout most of their range, *F. olivaceus* tends to occupy high gradient, clear streams, while *F. notatus* dominates more turbid backwater habitats of larger rivers and prairie streams (Howell and Black 1981). All three species in the *F. notatus* complex have a distinct lateral stripe that extends the length of the body along with black spots typically above the stripe. The number and intensity of these spots is cited as a diagnostic character for identification of *F. olivaceus* (Lee et al. 1980; Suttkus and Cashner 1981; Ross 2000). While spots of various colors are seen in a number of *Fundulus*, black spots are unique to the *F. notatus* complex. Thomerson (1966) noted variability in spot phenotypes across the range and hypothesized it may be a sexually di-morphic trait. In our own work with these species (Duvernell et al. 2007; Schaefer et al. 2009, 2011), we have noted substantial variation in *F. olivaceus* spot phenotypes among river systems and sexes and recognized that this trait is not always reliably diagnostic for species identification.

Our first objective was to quantify variability in the spot phenotype between males and females of the two more widespread species in the complex that display spots (*F. notatus* and *F. olivaceus*). We ask if the spot phenotype is sexually dimorphic and correlated with measures of condition and fitness for either species. Observed variability in the spot phenotype and results of the above analyses motivated us to ask detailed questions about the spot phenotype in *F. olivaceus*. Specifically, (1) do females select male *F. olivaceus* based on the spot phenotype? (2) is predation risk related to the spot phenotype? (3) what ecological variables are the best predictors of variability in the spot phenotype of *F. olivaceus* across its broad range? We assessed the efficacy of spot phenotype models at the individual, population, and river system level. Models included measures of key ecological traits hypothesized to be related to natural and sexual selection dynamics (predation pressure, habitat quality, and light environment).

## Methods

### Fish collection

Within each of the 13 river systems we identified between 8 and 12 sites (Table 1) for sampling. Based on museum records and earlier work with these species (Duvernell et al. 2007; Schaefer et al. 2011), sampling in each drainage was centered around the confluence of smaller tributaries (typical *F. olivaceus* habitat) and larger rivers (*F. notatus* habitat) with roughly half the sampling sites upstream in tributaries and downstream in larger rivers (Fig. 2). This ensured that both species and potential areas of coexistence were sampled along a gradient of stream sizes. At each site, we collected fish using a dip net and a 6.1 × 1.2 m seine with 0.3-cm mesh. Dip nets were used to specifically target *Fundulus* sp. that are easily visible at the surface and efficiently netted. Fish assemblages (including a smaller number of both species of *Fundulus*) were sampled by seining all available habitats over a 100–150 m reach. For each individual *Fundulus* sp. collected, a tissue sample was preserved for later genetic identification (Schaefer et al. 2011) before the fish was placed in an individually labeled 50-mL conical tube containing 10% formalin. Fish assemblage samples were preserved in 10% formalin and later transferred to 70% ethanol, identified to species, enumerated, and deposited into The University of Southern Mississippi Ichthyological Collection (http://ichthyology.usm.edu/usm/). At each site, we measured turbidity (NTU, HACH 2100 turbidity meter) and estimated canopy cover (mean of three estimates taken along transects at the upper, middle, and lower portions of sampled reach of stream).

**Table 1:** Number of sites sampled, number of adult (>32 mm SL) *Fundulus* analyzed, and a summary of the range of ecological variables measured at sites within each river system. Predation pressure is expressed as the log abundance of piscivorous fish and size represents cumulative drainage area. Names of the river systems as in Figure 1

River system	Sites	*F. notatus*	*F. olivaceus*	Predation (log abundance)	Turbidity (NTU)	Canopy (%)	Size (km^2^)
Amite	10	0	167	0.0–1.2	4.6–20.1	0.0–80.0	7–318
Black	9	9	202	0.3–1.1	1.7–52.4	1.7–52.4	32–21,397
Elk	7	35	82	0.5–0.8	7.2–20.7	10.0–80.0	61–3,97
Illinois	7	0	153	0.3–1.7	4.11–9.73	1.7–38.3	638–3840
Kiamichi	8	0	235	0.3–1.0	3.1–27.6	3.3–25.0	12–1806
Little	8	36	111	0.3–0.9	2.44–12.2	1.7–18.3	486–2996
Neches	8	53	117	0.0–0.6	9.4–24.2	0.0–73.3	45–20,500
Saline	12	102	160	0.0–1.1	1.5–189.0	0.0–80.0	4–498
Pascagoula	9	86	75	0.0–1.1	6.3–15.8	3.3–60.0	1738–21,177
Pearl	10	80	159	0.0–0.5	3.8–42.5	0.0–60.0	35–16,555
Sabine	9	5	101	0.3–0.8	6.2–123.0	0.0–48.3	45–21,530
Spring	10	42	165	0.0–0.8	5.9–26.1	3.0–35.0	337–6318
Tombigbee	10	72	113	0.6–1.2	3.8–14.0	1.7–70.0	31–6052

Totals	117	520	1840	0.0–1.7	1.5–189.0	0.0–80.0	4–21,530

**Figure 1:**
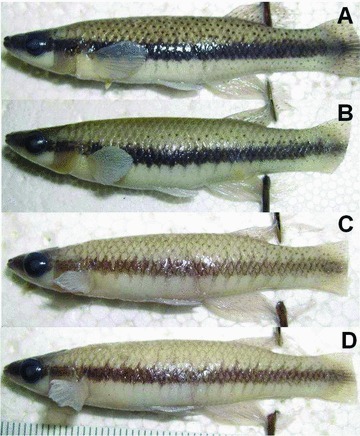
Photographs of large adult male *Fundulus olivaceus* ([A] from the Little River and [B] from the Saline River drainage) and *F. notatus* ([C and D] both from the Pearl River drainage) demonstrating the range of spot phenotypes seen in the species.

**Figure 2:**
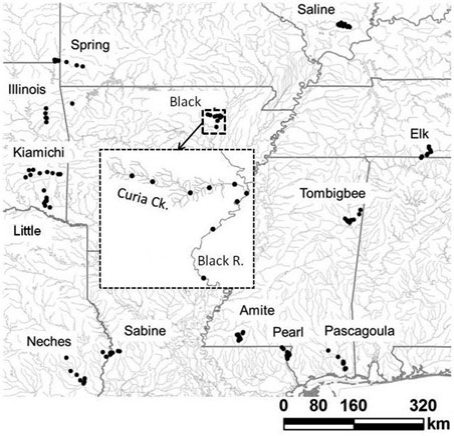
Map of sampling localities with each of the 13 river systems labeled. Within each system, sampling centered around a tributary-river confluence with equal numbers of sites upstream and downstream. Insert map shows the distribution of sampling sites within the Black River system in northeastern Arkansas.

### Spot density and individual condition

For each *Fundulus* sp., we counted the total number of visible dorsolateral spots on the left side of the body and measured standard length (SL), wet weight (mass), gonad mass, and body mass without gonads or gastrointestinal track (eviscerated mass). For analyses, we used only adults (>32 mm SL) for which sex could be determined reliably. Because the number of spots was positively correlated with SL (*r*^2^ = 0.43, *P* < 0.0001, df = 1,2573; spot count = 1.71 × SL – 21.5), we controlled for body size by dividing the number of spots by SL (hereafter spot density). To test and control for allometry, we first tested for it by regressing spot density and SL in each species-sex combination and then included SL as a covariate in analyses where appropriate. Body condition was assessed using Fulton's Condition Index (1000 × eviscerated mass/SL^3^) (Sutton et al. 2000) and reproductive condition assessed as gonadosomatic index (GSI, ratio of gonad mass to eviscerated mass). Individuals were assigned to species using diagnostic nuclear intron markers at five or more independent loci following Schaefer et al. (2011). Any individuals who were not homozygous at all five diagnostic loci were excluded from analyses. We used a two-way analysis of covariance (ANCOVA) (SL as covariate) to test for differences in spot density among species and sex. For each species and sex combination, we correlated spot density (SL as a covariate) with body condition and spot density with GSI to assess whether spot density was an indicator of condition or fitness.

### Spot density and male reproductive success

To assess the influence of spot density on reproductive success, we measured the proportion of offspring sired in laboratory mate choice trials. Male and female *F. olivaceus* were collected from creeks within the Pascagoula River drainage and housed in the University of Southern Mississippi wetlab facility. Males were initially screened at a single polymorphic microsatellite locus (FhATG-B103) (Adams et al. 2005; Duvernell et al. 2007; Gutierrez 2010). For each trial, four males with varying spot phenotypes whose offspring could be unambiguously identified with the single locus were selected, anesthetized, and photographed to count spots and measure SL. We calculated spot density as described above and ranked the four males in spot density. The four males and either one or two females were placed in 200-L round pools with gravel substrate and four acrylic mops in various locations of the pool that served as spawning media. Spawning mops were checked daily and a trial was considered complete when viable clutches were collected on four consecutive days. Eggs were incubated in smaller plastic cups for 10–20 days (Vigueira et al. 2008) and hatched larvae were preserved for genotyping after hatching. Trials produced a minimum of 10 eggs and when a larger number of eggs (80 or more in three trials) were produced roughly 25% of hatched larvae were selected at random for genotyping. After trials, males were again anesthetized and photographed to ensure spotting patterns did not vary significantly over the length of the trial. There was minimal variability in spot patterns and fish could easily be identified when comparing before and after photos. For a total of 13 trials (seven with one female and six with two females), we quantified the proportion of larvae sired by each of the four males. We used ANCOVA to test for differences (across all trials) in logit-transformed proportions of offspring sired among the four rank spot densities with SL as a covariate.

### Spot density and predation

To assess the influence of spot density on predation pressure, we measured survivorship when exposed to a predator (spotted bass, *Micropterus punctulatus*) (Knight and Gido 2005). Predation trials were conducted in a series of 24 outdoor mesocosms designed to mimic small stream habitat (Matthews et al. 2006). Mesocosms consisted of a pool (1.83 m diameter, 0.6-m deep with mixed sand/gravel substrate and woody debris as structure) and a riffle (0.9 m-long, 0.3-m wide, and 15-cm deep with gravel/cobble substrate) with recirculating pumps and a groundwater source. *Fundulus olivaceus* and *M. punctulatus* (135–210 mm SL) were collected from local tributaries in the Pascagoula River drainage and housed in three mesocosms (one for *M. punctulatus* and for each *F. olivaceus* sex) until trials began. For each trial, eight *F. olivaceus* (male and female trials done separately) were anesthetized and photographed to calculate spot density as described above. Because prey size is expected to strongly influence predator behavior, trials consisted of fish of similar sizes (mean SL of 54.5 mm for all trials, mean size range within trials was 4.5 mm with a maximum in one trial of 11 mm). Fish were introduced to mesocosms and allowed to acclimate for at least 30 min before a predator was introduced. Mesocosms were checked every 24 h to assess how many fish remained and trials ended when roughly half of the prey had been consumed (typically two to six days). Trials in which all fish were consumed within the first 24 h were excluded from analyses. The surviving fish were photographed and photos matched to pretrial photos. Six control trials were run with no predators to ensure that all fish were recovered and all mortality in experimental trials was due to predation. The eight fish in each trial were categorized as high or low spot density (four highest and lowest spot densities, respectively) and we tested for differences in logit-transformed survivorship between high and low spot density trials with paired *t*-tests for male and female trials.

### Individual, population, and drainage level variability in spot density

We used Akaike's Information Criteria (AIC_c_) for small samples size to compare the predictive power of candidate models for male and female *F. olivaceus* spot density at the individual, site, and drainage level (Anderson et al. 2000). Predictive variables (Table 2) included predation pressure (log abundance of all piscivorous species collected [*Lepisosteus* sp., *Micropterus* sp., *Esox* sp., *Lepomis cyanellus*, and *L. gulosus*]), water clarity (turbidity), reproductive investment (GSI), condition (Fulton's condition index), canopy cover, and drainage (river system). For individual male and female models, condition and GSI represented measures of individual fitness. For population level models, GSI and condition were averaged for each sex at each site and served as a measure of habitat quality. For river system models of spot density, these variables along with predation pressure, canopy cover, and turbidity were averaged across all sites within each river system. The drainage variable was not included in river system models. Candidate models included a null (no variables), each one of these variables alone and all possible combinations of two-way models. Individual, site or drainage mean SL was included in all models except for the null to control for allometry. Three way and higher interactions were not included in analyses due to difficulties with interpretation. Models with low dAIC_c_ and high Akaike weights (*w_i_*) have the best combination of parsimony (few parameters) and predictive power. We only interpreted models with dAIC_c_ <2.0 and *w_i_* score >10% of the highest *w_i_* score as meaningful (Anderson and Burnham 2002). All analyses were performed in the R statistical language (R Development Core Team 2010).

**Table 2:** Variables used in AIC modeling of individual, population, and river system level models of male and female spot density in *Fundulus olivaceus*. For individual models, individual reproductive investment (GSI) and condition (Fulton's condition index) values were used. For population and river system level models, those variables were averaged as indicators of habitat quality. Other site-specific variables (predation pressure, water clarity, and canopy cover) were averaged for river system models. At each of the three levels, candidate models included all variables alone and all possible combinations of two-variable models with interaction terms. Standard length (individual, site, or drainage mean) was a covariate in all models to control for allometry

Variable	Measure	Hypotheses
Predation pressure	Log abundance of piscivores in local fish community	Increased predation pressure selects against increased spot density.
Reproductive investment or habitat quality	Gonadosomatic index	Spot density is an indicator of fitness on the individual level. On the site (population) and drainage level, more favorable habitat will have healthier fish that have larger gonads and display more spots.
Condition or habitat quality	Fulton's condition index	Spot density is an indicator of condition on the individual level. On the site (population) and drainage level, more favorable habitat will have healthier fish that are in better condition and display more spots.
Water clarity	Turbidity (NTU)	Decreased water clarity reduces sexual selection for increased spot density.
Canopy cover	Canopy cover (%)	Decreased light availability reduces sexual selection for increased spot density.
Drainage	13 river systems sampled	Spot density is not under strong selection and patterns will best be explained by random differences among drainages (drift).

## Results

### Variability in spot density among sex and species

**Figure 3:**
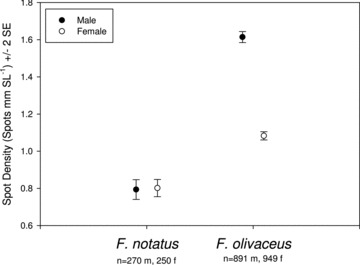
Mean (±2 SE) spot density for male and female *Fundulus notatus* and *F. olivaceus*.

We collected data on 2575 adult *Fundulus* from 117 sites in the 13 river systems. The number of spots ranged from 0 (several male and female *F. notatus* from the Pascagoula River) to over 150 (male *F. olivaceus* from tributaries in the Spring and Black River systems) (Fig. 1). Spot densities showed positive allometry for male *F. olivaceus* (SL-spot density slope = 0.021, *r*^2^ = 0.213, *P* < 0.001) and negative allometry for female *F. olivaceus* (slope = –0.003, *r*^2^ = 0.008, *P* < 0.001) and male *F. notatus* (slope = –0.012, *r*^2^ = 0.046, both *P* < 0.001), but no pattern of allometry in female *F. notatus* (slope = –0.005, *r*^2^ = 0.009, both *P* < 0.146). There was a significant difference in spot density among the two species (*F. olivaceus* mean = 1.34 ± 0.011 SE; *F. notatus* mean = 0.798 ± 0.018 SE; *F*_1,2355_ = 74.3, *P* < 0.001, η_p_^2^ = 0.22) and an interaction (*F*_1,2355_ = 223.7, *P* < 0.001, η_p_^2^ = 0.07) between species and sex (Fig. 3). This interaction was due to male *F. olivaceus* having significantly higher spot density than females (*F*_1,1837_ = 122.6, *P* < 0.001, η_p_^2^ = 0.31) while the sexes did not differ in *F. notatus* (*F*_1,517_ = 0.07, *P* < 0.79, η_p_^2^ < 0.01).

### Spot density and individual condition

**Figure 4:**
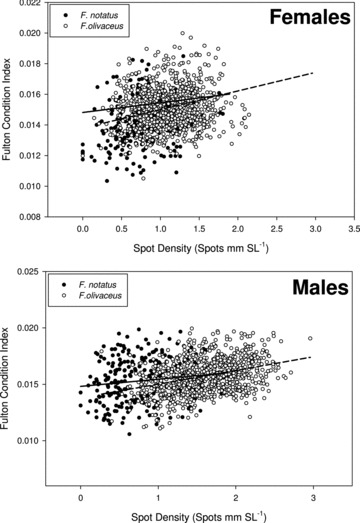
Relationship between spot density and Fulton's condition index in female (top panel) and male (bottom panel) *Fundulus notatus* and *F. olivaceus*. Lines represent best fit linear regressions.

**Figure 5:**
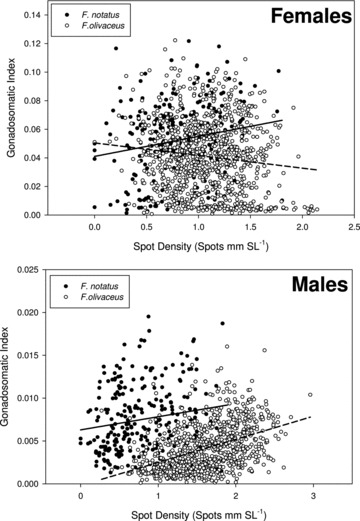
Relationship between spot density and gonadosomatic index in female (top panel) and male (bottom panel) *Fundulus notatus* and *F. olivaceus*. Lines represent best fit linear regressions.

Spot density was positively correlated with condition in male *F. olivaceus* (*F*_1,889_ = 23.7, *P* < 0.001, *r*^2^ = 0.23) and male *F. notatus* (*F*_1,268_ = 24.62, *P* < 0.001, *r*^2^ = 0.13). Spot density was positively correlated with condition in female *F. olivaceus* (*F*_1,947_ = 31.69, *P* < 0.001, *r*^2^ = 0.04) and female *F. notatus* (*F*_1,248_ = 20.7, *P* < 0.001, *r*^2^ = 0.12) (Fig. 4). Spot density was positively correlated with GSI in male *F. olivaceus* (*F*_1,889_ = 96.70, *P* < 0.001, *r*^2^ = 0.27) and male *F. notatus* (*F*_1,268_ = 20.70, *P* < 0.001, *r*^2^ = 0.12) (Fig. 5). Spot density was negatively correlated with GSI in female *F. olivaceus* (*F*_1,947_ = 8.90, *P* < 0.001, *r*^2^ = 0.02) but positive in female *F. notatus* (*F*_1,248_ = 16.90, *P* < 0.001, *r*^2^ = 0.08).

### Reproductive success and predation pressure

**Figure 6:**
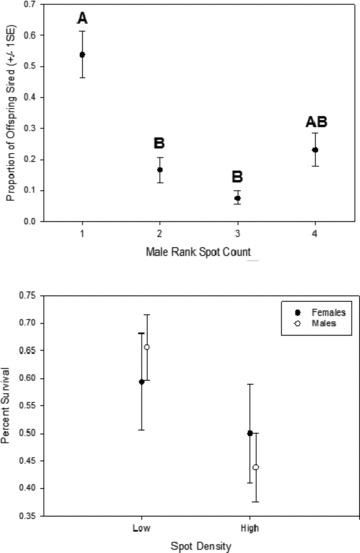
Proportion of offspring sired by rank male spot density (top panel) and mean survival rate for males and females with high or low spot densities.

Within mate choice trials, the range (difference between first and fourth ranked individuals) of spot densities for males averaged 0.68 (minimum of 0.31, maximum of 1.10). There was a significant difference in male reproductive success among the four spot density ranks (*F*_3,47_ = 3.39, *P* < 0.025, η_p_^2^ = 0.16). Males that ranked first in spot density sired on average 53.8% of offspring that was significantly greater than males that ranked second (15.4%, Tukey multiple comparison *P* < 0.021) or third (7.6%, *P* < 0.006), but not different than males that ranked fourth (23.1%, *P* < 0.063). There were no pairwise significant differences in male reproductive success among second, third, or fourth ranked males (Fig. 6A). Within predation trials, the range (difference between first and fourth ranked individuals) of spot densities averaged 0.82 (minimum of 0.31, maximum of 1.31). The rate of survival in predation trials was significantly lower for high spot density males compared to low spot density males (43.8 vs. 65.6% survival, *t* = –2.73, *P* < 0.016). There was no difference in survivorship between high and low spot density females (50.0 vs. 59.4%, *t* = –0.38, *P* < 0.72) (Fig. 6B).

### Models of *F. olivaceus* individual, population, and river system spot density

**Figure 7:**
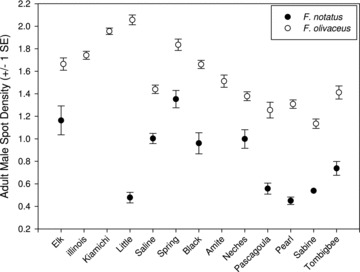
Mean spot density (±1 SE) by river system for mature male *Fundulus notatus* and *F. olivaceus*.

**Figure 8:**
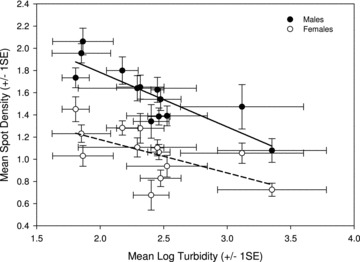
Relationship between mean turbidity and spot density among the 13 river systems for adult male and female *Fundulus olivaceus*. Values are the means among all sites in each system ±1 SE.

Individual spot density was best predicted by a single model in male (Drainage × GSI) and female (Drainage × Canopy; Table 3) *F. olivaceus*. Both models contained drainage as a variable and predicted between 39% and 53% of the variability among individuals. On a population level, drainage was again a component of both interpretable models. Average male spot density at sites was best predicted by a single model (Drainage) and average female spot density was best predicted by the Drainage × GSI model. Differences among drainages were substantial with nearly 63% of the variation at the site level explained by drainage differences (Table 3; Fig. 7). On the river system scale, both male and female mean spot density was best predicted by Turbidity alone and for females the Turbidity × GSI model was also interpretable. Across drainages, Turbidity alone was a better predictor of male than female spot density (Table 3), but the slopes of the relationship for males and females were very similar (Fig. 8).

**Table 3:** Model complexity (K), AIC_c_ scores, criteria for model selection (dAIC_c_, *w*_i_), and adjusted *r*^2^ for individual, population, and river system level models of male and female spot density in *Fundulus olivaceus*. Only interpretable models (dAIC_c_ <2.0 and *w_i_* score >10%) are presented. Standard length was a covariate in all models to control for allometry

Model	K	AIC_c_	dAICc	W*_i_*	*r*^2^
Individual					
Male *F. olivaceus*					
Drainage × GSI	28	455.2	0.0	0.99	0.533
Female *F. olivaceus*					
Drainage × Canopy	28	230.1	0.0	0.98	0.392
Population					
Male *F. olivaceus* site means					
Drainage	15	7.5	0.0	0.987	0.626
Female *F. olivaceus* site means					
Drainage × GSI	27	725.0	0.0	0.497	0.615
River system					
Male *F. olivaceus*					
Turbidity	4	–3.3	0.0	0.892	0.744
Female *F. olivaceus*					
Turbidity	4	101.0	0.0	0.549	0.537
Turbidity × GSI	5	101.8	0.8	0.377	0.771

## Discussion

Patterns of variability in the spot phenotype for *F. olivaceus* were consistent with the handicap principle. In *F. olivaceus*, spot density was an indicator of male fitness and females preferred males with more spots in mate choice trials involving fish from the Pascagoula River drainage. Predation pressure as a putative cost to higher spot density was supported in mesocosm predation trials (fish from the Pascagoula River drainage) but not in field data as predator abundance was not part of interpretable models at any level. In many well-studied systems where predation pressure is consistently shown to influence male phenotypes (Rosenthal et al. 2001; Langerhans et al. 2004; Millar et al. 2006), predation pressure is effectively depicted as a dichotomous presence or absence (Krebs and Bell 2011). In this system, predation is more complex as predators (large-bodied piscivores) were in all likelihood ubiquitous. The sampling gear used (seine) has been shown to preferentially select small-bodied species (Gido et al. 2009), meaning we likely undersampled predators, especially in larger river habitats. The expectation (Vannote et al. 1980) is that the abundance and size of predators will increase with increasing stream size. Turbidity is expected to (and did) follow the same pattern of increasing with stream size within a river system. Thus, it is difficult in some respects to disentangle the potential influence of turbidity and predation pressure within river systems in this study. However, there were clear differences in turbidity among river systems that correlated strongly with river system level differences in spot density. There is no reason to expect (from our data or what is known of North American fish community composition) significant drainage level differences in predator abundance (Matthews 1998).

Assuming predation pressure is generally consistent across river systems and the abundance of large-bodied piscivores increases in downstream habitat (Vannote et al. 1980), there are a number of indicators that predation has played an important role in the evolution of *F. notatus* and *F. olivaceus*. The expectation (Reznick and Endler 1982; Reznick et al. 2001; Krebs and Bell 2011) is that predation pressure will select for a suite of life-history traits that are consistent with observed differences between these two species. *Fundulus notatus* (higher predation habitats) is smaller (Lee et al. 1980), reproduces earlier at a smaller size (J. F. Schaefer, unpubl. data), produces a larger number of smaller offspring (Vigueira et al. 2008) and invests more energy in reproduction (Fig. 4). It is also intriguing that within *Fundulus*, the black spot phenotype is unique to these species and evolved as a sexually dimorphic trait in upstream habitats that feature clearer water and presumably lower predation pressure.

### Genetic versus plastic contributions

We do not have data to directly address the relative contribution of genetic and environmental influences on the spot phenotype. While both mechanisms are likely involved with expression of this trait (Fuller and Travis 2004), a number of observations would be consistent with a fairly limited adult plasticity. First, spot phenotypes did not change over short periods of time (4–21 days) in predation (conducted in an outdoor setting with natural food, light, and photoperiod) or mate choice (conducted indoors with artificial food and lighting) experiments. In all cases, spot patterns acted like a fingerprint and individual fish could be unambiguously matched through time. For predation trials, we had two authors independently match individuals using the same digital images. Second, spot phenotypes (unlike fin color) are generally not seasonally variable. While not quantified, our experience working with these species is that individuals caught in early spring or late fall (outside the breeding season) still express the spot phenotype. This general consistency in adult spot patterns is likely how this trait came to be regarded as diagnostic (Suttkus and Cashner 1981) among the species. While this indicates that adult phenotypes may be somewhat fixed, the trait clearly changes allometrically (see above) and there may be a considerable developmental plasticity (West-Eberhard 2003) component. A heritability study is planned to directly address the genetic contribution to this phenotype.

### Natural and sexual selection

The exact mechanisms that lead to either increased predation or reproductive success of higher spot density fish is not known. Species in the *F. notatus* species complex are counter shaded (unpigmented below and darker above) and the broad lateral stripe extending from the tail through the eye (unique among *Fundulus*) is most likely an example of disruptive coloration (Burtt 1981; Stevens and Merilaita 2009). Disruptive color patterns feature areas of high contrast to break up body shape in which case high spot density may make males more conspicuous by reducing contrast or outlining the body. It is also possible that the spots are not the direct cause of increased predation risk. Larger reproductive investment may merely be correlated with spot density while behavioral changes increase exposure to predators and reproductive success. In documenting mating behaviors, substantial male–male and even female–female aggression was observed along with male lateral displays to females (Gutierrez 2010). The potential importance of male dominance was also evident in two mate choice trials where one of the successful males was a substantially larger fish with relatively few spots (ranked fourth in spot density). We did not collect behavioral data as part of these trials, but our results are consistent with females preferring size and high spot density or male–male dominance resulting in larger males having more access to females (Fig. 4). It should also be pointed out that the arenas where mate choice trials took place were fairly small (1-m diameter pools), which would exacerbate the importance of aggressive interactions. Regardless of the mechanisms involved, females preferred increased spot density that clearly had a fitness cost to males in the form of increased predation risk.

### Geographic patterns

One of the more appealing aspects of this experimental system is the species broad distribution across ecologically disparate river systems (Schaefer et al. 2011). Differences among river systems were clearly important (Fig. 6), as the drainage variable was part of every interpretable spot density model at the individual and population level (Table 3). Differences among river systems were not random (expectation if selective pressures were weak) as turbidity was strongly correlated with spot phenotypes on the river system level (Table 3; Fig. 7). The general pattern revealed was that drainages in the Coastal Plain (right side of *x*-axis in Fig. 6, Amite, Neches, Pascagoula, Pearl, Sabine, and Tombigbee) have lower spot densities than those in the Ozark (Illinois, Black, and Spring), Ouachita (Little and Kiamichi), or Eastern Highlands (Elk). The lone river system outside of the Coastal Plain with low spot density is in the Saline River, a tributary of the Ohio River in the formerly glaciated Southern Till Plain of southern Illinois. The Ozark, Ouachita, and Eastern Highland tributaries (*F. olivaceus* habitat) typically feature larger (cobble and gravel) and more stable substrate compared to prairie or coastal systems where tributaries typically have finer gravel, sand, silt, or clay substrate. These differences likely result in observed turbidity patterns across river systems. We can also generally rule out phylogeographic influence as the cause of river system differences in spot density. A population level phylogeny (B. R. Kreiser, unpubl. data) has revealed relatively little structure across the range for *F. olivaceus* (distinctly different from patterns in *F. notatus*). Even with modest grouping of coastal versus inland populations (supported by phylogeography, B. R. Kreiser, unpubl. data), relationships between turbidity and spot density persist within these two groups.

While spot expression in male *F. olivaceus* fits predictions of the handicap principle, females also express the phenotype. In many well-studied sexually dimorphic fishes, genes controlling dimorphic color traits are often linked to the Y-chromosome and not expressed in females (Lindholm and Breden 2002; Gordon et al. 2011). Female expression of spots raises the possibility that the spot phenotype in these fish has a different genetic architecture or control mechanism. One possibility is hormonal regulation, something common in sexually dimorphic color patterns (Parker et al. 2011). Haskins et al. (1961) found that testosterone treatments led to the expression of non-Y-linked color genes in female guppies. Hormonal regulation of spot density in *F. olivaceus* would be consistent with the observed strong male allometry (spot density increases as males mature) and the negative correlation between female spot density and GSI (Fig. 5) but not female condition (Fig. 4). The similarity in male and female spot densities across drainages (Fig. 8) is intriguing as a signal of parallel evolution of the sexes within a species, something generally not well studied (Hendry et al. 2006). As a whole, our data are consistent with natural and sexual selection acting on males driving parallel evolution of both sexes in a trait that is hormonally regulated.

Data for this study were compiled over distinct linear gradients within a broad range of disparate river systems. The result is compelling support for some fundamental processes in evolutionary ecology. Future work with this system is planned to address the underlying genetic framework and heritability of the spot phenotype and other suspected sources of variability (such as plasticity). The exact role of spots as a reproductive isolating mechanism among the species has not been tested but one would suspect some role based on mate choice data presented. The potential interaction between the local environmental gradients, expression of traits contributing to reproductive barriers, and the structure of contact zones (Schaefer et al. 2011) provides ample opportunity to address a number of stimulating questions.
